# Fatigue Life Prediction of Notched Details Using SWT Model and LEFM-Based Approach

**DOI:** 10.3390/ma16051942

**Published:** 2023-02-26

**Authors:** Rui Hao, Zongyi Wen, Haohui Xin, Weiwei Lin

**Affiliations:** 1 Department of Civil Engineering, Aalto University, 02150 Espoo, Finland; 2 Department of Bridge Engineering, Southwest Jiaotong University, Chengdu 610021, China; 3 Department of Civil Engineering, School of Human Settlements and Civil Engineering, Xi’an Jiaotong University, Xi’an 710049, China

**Keywords:** fatigue, XFEM, numerical simulation, fatigue life prediction, UDMGINI

## Abstract

The fatigue crack initiation life of unwelded steel components accounts for the majority of the total fatigue life, and the accurate prediction of it is of vital importance. In this study, a numerical model utilizing the extended finite element method (XFEM) and Smith–Watson–Topper (SWT) model is established to predict the fatigue crack initiation life of notched details extensively used in orthotropic steel deck bridges. Using the user subroutine UDMGINI in Abaqus, a new algorithm was proposed to calculate the damage parameter of SWT under high-cycle fatigue loads. The virtual crack-closure technique (VCCT) was introduced to monitor crack propagation. Nineteen tests were performed, and the results were used to validate the proposed algorithm and XFEM model. The simulation results show that the proposed XFEM model with UDMGINI and VCCT can reasonably predict the fatigue lives of the notched specimens within the regime of high-cycle fatigue with a load ratio of 0.1. The error for the prediction of fatigue initiation life ranges from −27.5% to 41.1%, and the prediction of total fatigue life has a good agreement with the experimental results with a scatter factor of around 2.

## 1. Introduction

Fatigue life prediction is of vital importance for structures and components subjected to repeated loading. Generally, the fatigue life comprises two parts, crack initiation life and crack propagation life. For welded joints, a massive part of the fatigue life accounts for the crack propagation life [1]. Nevertheless, for the unwelded components or structural details, the fatigue crack initiation life takes a larger part of the total fatigue life as the initial defects are less detrimental than that of welded ones. As a typical instance among unwelded structural details, the notch detail is widely employed in civil engineering, e.g., the cutout in the rib-to-floor beam connections of orthotropic steel deck (OSD) bridges [2,3]. Unlike the fatigue mechanism of welded details, the fatigue damage at the cutout is caused by the geometrical discontinuity within a localized area. As a result, it remains challenging to apply the traditional nominal-stress-based method to evaluate the fatigue life of notch details subjected to complex stress states [4]. To overcome the disadvantages of the nominal-stress method, other evaluation methods, including local stress–strain methods, the theory of critical distance (TCD), and weighting-control-parameter-based methods, have been investigated by scholars in recent decades [5,6,7]. Different from the nominal-stress method, the local stress–strain methods take the maximum stress and strain at the notch root as the indicator of the fatigue assessment [8]. Rather than taking the peak stress, Neuber [9] proposed a more reasonable way that takes the averaged stress over a certain zone as the indicator, which now is known as TCD. TCD correlates the fatigue life with the equivalent stress range that is averaged over a zone determined by the critical distance [10]. Similar to TCD, the weighting-control-parameter-based methods consider that the fatigue damage is governed by the mechanical response within a local zone, and define the control parameters by stress/strain [11] or strain energy [12]. However, these methods also have some drawbacks. For example, the local stress–strain methods generally underestimate the fatigue life of components [5], and TCD requires two calibration S-N curves obtained in experimental fatigue tests to calibrate the relevant parameters, which increases the cost [7]. To achieve a more accurate fatigue life prediction, taking full advantage of the developed high-performance computing, i.e., performing numerical simulation, is a preferable alternative compared to experimental fatigue tests. However, well-built standard procedures for fatigue life prediction in both the high-cycle fatigue (HCF) regime and low-cycle fatigue (LCF) regime are still insufficient.

Inspired by Wöhler’s pioneer work on correlating the applied stress range to the fatigue life (also known as S-N curve), different types of fatigue damage models were then developed, and they can be roughly classified as stress-based models, strain-based models, energy-based models, and critical plane models [13]. Along with the adoption of S-N curves in fatigue evaluation, stress-based models are widely employed in many standards, such as Eurocode 3 [14], and the most frequently used one is the Basquin equation [15] shown in Equation (1),

(1) $$\frac{\Delta \varepsilon_{e}}{2}=\frac{{\sigma^{\prime }}_{f}}{E}{\left(2N_{f}\right)}^{b}=\frac{\Delta \sigma_{e}}{2E}$$

where Δ*ε_e_* is the elastic strain range, *σ*′*_f_* is the fatigue strength coefficient that is material-related, *E* is the elastic modulus of steel, *b* is the fatigue strength exponent, and *N_f_* is the fatigue life. It describes the relationship between stress range and fatigue life on a log–log scale. Based on this model, many other models accounting for the mean stress effects were later proposed, such as Goodman, Gerber, and Soderberg relations [16], Manson–McKnight models [17], etc.

In LCF cases, considerable plastic strain is generated, and stress-based models are apparently not qualified to yield satisfactory accuracy, resulting in the development of the strain-based models. The basic idea of strain-based models is that the fatigue life is controlled by the plastic strain range. The Coffin–Manson equation [18], shown in Equation (2), was first proposed to depict the relationship between the plastic strain range and fatigue life.

(2) $$\frac{\Delta \varepsilon_{p}}{2}={\varepsilon^{\prime }}_{f}{\left(2N_{f}\right)}^{c}$$

where Δ*ε_p_* is the plastic strain range, *ε*′*_f_* is the fatigue ductility coefficient that is material-related, and *c* is the fatigue ductility exponent. Later on, the Coffin–Manson equation was modified as the Morrow equation [19] to extend its applicability in both HCF and LCF regimes.

(3) $$\frac{\Delta \varepsilon }{2}=\frac{{\sigma^{\prime }}_{f}}{E}{\left(2N_{f}\right)}^{b}+{\varepsilon^{\prime }}_{f}{\left(2N_{f}\right)}^{c}$$

where Δ*ε* is the total strain range.

The critical plane models, which define the critical plane as where the most severe damage is likely to occur, assume that fatigue damage is governed by functions of either only stress/strain or the product of stress and strain on the critical plane. Normal stresses and strains are hypothesized to open the fatigue crack, while shear stresses and strains produce dislocation along slip lines, driving the initiation and propagation of cracks [13]. When the effect of the mean stress is not neglectable, the most frequently used model is the Smith–Watson–Topper (SWT) damage model [20] (Equation (4)), in which the function of the product of the maximum normal stress and strain amplitude is hypothesized to govern the fatigue life.

(4) $$\frac{\sigma_{\max}\Delta \varepsilon }{2}=\frac{{\left({\sigma^{\prime }}_{f}\right)}^{2}}{E}{\left(2N_{f}\right)}^{2b}+{\sigma^{\prime }}_{f}\cdot {\varepsilon^{\prime }}_{f}{\left(2N_{f}\right)}^{b+c}$$

where *σ*_max_ is the maximum normal stress on the critical plane, and Δ*ε* is the range of principal strain. This model is essentially a derivation from the manipulation of Equations (1) and (3). By incorporating the SWT model into the finite element model, Xin [21] conducted a pilot study on the numerical prediction of the fatigue crack initiation life of smooth steel specimens. Fatemi and Socie [22] proposed a damage model considering the maximum shear strain range to be the primary damage driver on the critical plane and the maximum normal stress normalized by the yield stress. Glinka et al. [23] proposed a parameter that multiplies the shear strain amplitude and normal stress amplitude on the critical plane. In addition, many energy-based models were also proposed. The energy-based models correlate fatigue life with strain energies. Liu [24] proposed a virtual strain energy model that considers both the tensile failure mode and shear failure mode. Chu et al. [25] included the effect of mean stress by replacing the stress ranges with maximum stresses. The aforementioned Glinka–Wang–Plumtree model [23] was proposed when they noted that the Chu–Conle–Bonnen model may ignore the effect of mean stress in a nonproportional loading condition when the strain range equals 0 [26]. For the simulation of strong geometrical discontinuities such as cracks, the traditional finite element method encounters several tricky problems when tackling cracks. For instance, adaptive remeshing is necessary for every increment when the crack propagates, and the mesh size near the crack tip is required to be fine enough to accurately predict the stress field and stress intensity factors. The extended finite element method (XFEM) provides a solution by adding additional degrees of freedom to the elements within the region where the possible crack path goes through, or the so-called enriched region. For XFEM, the remeshing is unnecessary and the crack tip element is not required to be refined, making it a potential tool to simulate the fatigue procedure.

Few studies are available for the numerical prediction of fatigue crack life incorporated with the SWT model. Though the study [21] successfully validates the numerical model with smooth specimens under tension–compression fatigue loading, the fatigue cycle increment has to be specified manually. To investigate the total fatigue life of the notch detail and extend the applicability of the SWT-model-based numerical simulation, in this study, a new algorithm considering the cycle-by-cycle fatigue damage accumulation is proposed and implemented into the XFEM model by using the user-defined subroutine UDMGINI without the necessity to specify the fatigue cycle increment, and the model is validated against test results of notched specimens under tension–tension loading. Moreover, a linear elastic fracture mechanics (LEFM) approach is also used for fatigue propagation analysis. The new algorithm is validated against the experimental results of several groups of notched specimens. This paper consists of the following sections: Section 2 explains the methods used in the numerical model to predict total fatigue life; Section 3 describes the numerical model and the fatigue test that were used to validate the model; and Section 4 presents the obtained results and relevant discussion.

## 2. Numerical Implementation of Fatigue in Abaqus

The fatigue life of structural details consists of two parts: fatigue crack initiation life and propagation life. For the crack initiation life, the subroutine UDMGINI is used to implement the SWT fatigue damage model, which is presented in Section 2.1. The crack propagation life is simulated by the virtual crack-closure technique (VCCT), which is a LEFM-based approach shown in Section 2.2.

### 2.1. Algorithm for the UDMGINI Subroutine

As there is no built-in damage initiation model that is suitable for the prediction of fatigue failure, the user subroutine UDMGINI provided by Abaqus [27] is employed to customize the user-defined damage initiation criterion based on the SWT model. UDMGINI will insert a “crack” into the enriched elements when the user-specified criterion is met, and the “crack” is described by the enriched degrees of freedom (DOFs) in XFEM. In the formulation of XFEM [28], the displacement ***u***(***x***) of any integration points in the element can be expressed by Equation (5),

(5) $$u\left(x\right)=\sum_{i=1}^{N}N_{i}\left(x\right)u_{i}+\sum_{j=1}^{M}N_{j}\left(x\right)H\left(x\right)a_{j}$$

where:
*N*is the number of nodal points in a finite element;*N_i_* (***x***)is the standard shape function;***u**_i_*is the standard nodal displacement related to the standard shape function;*M*is the number of enriched nodes in a finite element;*N_j_* (***x***)is the shape function of the enriched part;***a**_j_*is the nodal DOF corresponding to the enrichment function;*H* (***x***)is the Heaviside enrichment function, defined as Equation (6).

(6) $$H\left(x\right)=\left\{ \begin{array}{l} 1,\;\mathrm{if}\;\varphi \left(x\right)=\left\|x-x^{*}\right\|\mathrm{sign}\left(n_{\Gamma_{d}}\cdot \left(x-x^{*}\right)\right)\geq 0\\ -1,\;\mathrm{if}\;\varphi \left(x\right)=\left\|x-x^{*}\right\|\mathrm{sign}\left(n_{\Gamma_{d}}\cdot \left(x-x^{*}\right)\right)<0\; \end{array}\right.$$

where:
*φ* (***x***)is the signed distance function;x*is the closest point projection of ***x*** onto the crack surface Γ*_d_*;**n**_Γd_is the normal vector to the crack surface at point x*.

Currently, the subroutine UDMGINI is only available for enriched elements [27]. Based on the SWT model, a fatigue damage factor *d_f_* [21] is defined in Equation (7) and its value determines the parameter FINDEX in the subroutine UDMGINI. When the damage factor accumulates to a value of 1.0, the parameter FINDEX is set to 1.0 and then a fatigue crack is inserted into the critical element on the dominant failure plane specified in the SWT model, i.e., the plane where the maximum principal strain is found.

(7) $$d_{f}=\frac{\frac{\sigma_{\max}\Delta \varepsilon }{2}}{\frac{{\left({\sigma^{\prime }}_{f}\right)}^{2}}{E}{\left(2N_{f}\right)}^{2b}+{\sigma^{\prime }}_{f}\cdot {\varepsilon^{\prime }}_{f}{\left(2N_{f}\right)}^{b+c}}$$

To enable a numerical calculation, the partial derivative of *d_f_* with respect to *N_f_* (see Equation (8)) is used to account for the incremental variation of fatigue damage with the increasing of loading cycles.

(8) $$\frac{\partial d_{f}}{\partial N_{f}}=-\frac{\sigma_{\max}\Delta \varepsilon \left[2b\frac{{\left({\sigma^{\prime }}_{f}\right)}^{2}}{E}{\left[2\left(N_{f}+\Delta N_{f}\right)\right]}^{2b-1}+\left(b+c\right){\sigma^{\prime }}_{f}\cdot {\varepsilon^{\prime }}_{f}{\left[2\left(N_{f}+\Delta N_{f}\right)\right]}^{b+c-1}\right]}{2{\left[\frac{{\left({\sigma^{\prime }}_{f}\right)}^{2}}{E}{\left[2\left(N_{f}+\Delta N_{f}\right)\right]}^{2b}+{\sigma^{\prime }}_{f}\cdot {\varepsilon^{\prime }}_{f}{\left[2\left(N_{f}+\Delta N_{f}\right)\right]}^{b+c}\right]}^{2}}$$

After the derivation of the partial derivative, Equation (9) is used to calculate the accumulated fatigue damage Δ*d_f_* in the loading cycle Δ*N_f_*.

(9) $$\Delta d_{f}=\frac{\partial d_{f}}{\partial N_{f}}\Delta N_{f}$$

**Figure 1:**
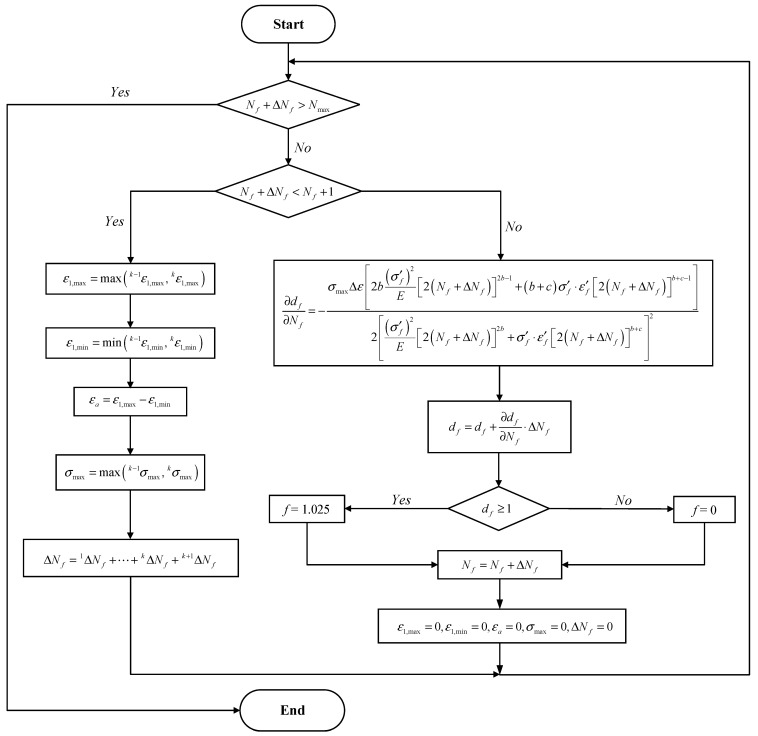
Flowchart of the fatigue damage initiation model.

To calculate the incremental fatigue damage in each cycle, a new algorithm considering the cycle-by-cycle fatigue damage accumulation is proposed, and Figure 1 shows the workflow of the implementation of the SWT-based fatigue damage model. Since the fatigue damage is accounted cycle-by-cycle, the incremental loading cycle Δ*N_f_* is a value close to 1 (assuming the period of the cyclic load is 1).

In this study, since there is no experimental data available for calibrating the material constants in the SWT model, the corresponding values from literature are utilized. Table 1 shows the material constants in the SWT damage parameter calibrated by Cruces [29]. The Cruces’ parameters are used in this study.

### 2.2. LEFM-Based Fatigue Crack Propagation Analysis

**Figure 2:**
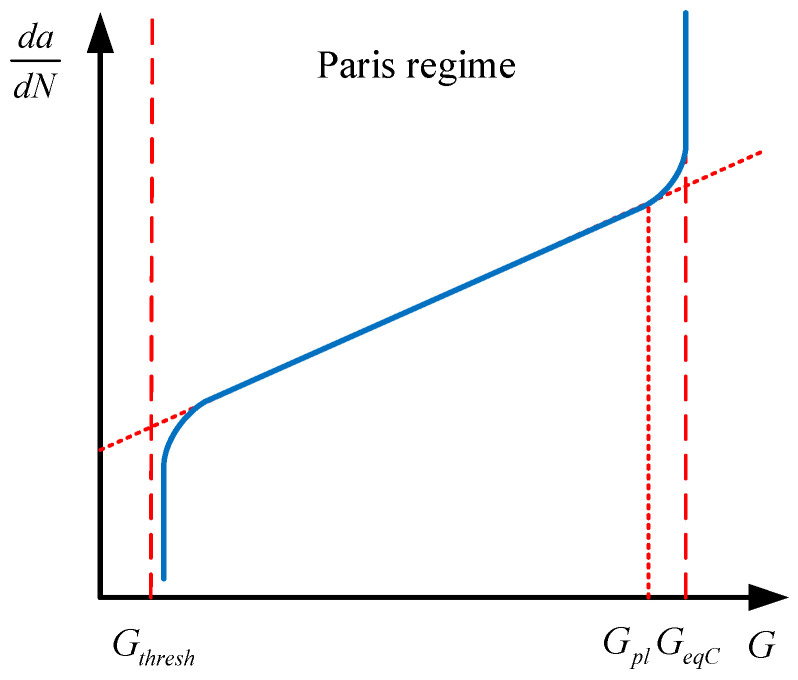
Paris’ law in terms of energy.

Once the fatigue crack initiates, Equation (10) is used as a criterion for fatigue crack propagation analysis,

(10) $$\frac{da}{dN}=c_{3}\Delta G^{c_{4}}$$

where *c*_3_ and *c*_4_ are the material constants in Paris’ law in terms of fracture energy, and the values of these two constants are taken as 1.4 × 10^−5^ and 1.8121 according to the experimental results in [30]. Equation (10) relates the relative fracture energy release rate Δ*G* to the fatigue crack propagation rate *da*∕*dN*, as shown in Figure 2. When the Δ*G* is larger than the energy release rate threshold *G_thresh_*, but no larger than the energy release rate upper limit *G_pl_*, the crack will propagate following Equation (10). The determination of the equivalent fracture energy release rate *G_eqC_* is based on the user-selected mix mode, which is the power law expressed by Equation (11) in this study. For the determination of the energy release rate threshold *G_thresh_* and the energy release rate upper limit *G_pl_*, the ratio *G_thresh_* ∕ *G_eqC_* of 0.1 and *G_pl_* ∕ *G_eqC_* of 0.9 are specified, respectively [27]. The critical energy release rates for three fracture modes *G_IC_*, *G_IIC,_* and *G_IIIC_* are 6.5, 6.5, and 6.5 N/mm, respectively, and the corresponding exponents *a_m_*, *a_n_*, and *a_o_* for them are 1, 1, and 1, respectively [30], as listed in Table 2. The fracture energy release rates at the crack tips in the enriched elements are calculated based on VCCT.

(11) $$\frac{G_{eq}}{G_{eqC}}={\left(\frac{G_{I}}{G_{IC}}\right)}^{a_{m}}+{\left(\frac{G_{II}}{G_{IIC}}\right)}^{a_{n}}+{\left(\frac{G_{III}}{G_{IIIC}}\right)}^{a_{o}}$$

**Figure 3:**
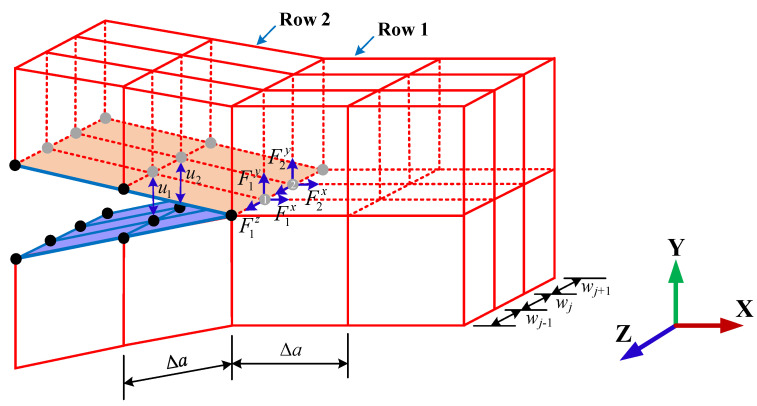
Illustration of VCCT.

VCCT is established based on two assumptions [31]: (1) the released fracture energy ∆*G* on the extended crack surface ∆*a* ∙ *w_j_* equals the required energy that can exactly close the extended crack, and (2) the stress field near the crack tip approximately remains the same before and after a small crack extension of ∆*a*. As shown in Figure 3, according to VCCT, the required energy ∆*G* for the elements in Row 1 to extend for ∆*a* is the nodal forces on the crack front multiplied by the nodal displacements of the elements in Row 2. The expression of the calculation of *G* in the three fracture modes are shown in Equations (12)–(14). However, for the XFEM-enriched elements, the nodal displacements are obtained on the enriched DOFs rather than the standard FEM nodes.

(12) $$G_{I}=\frac{1}{2\Delta a\cdot w_{j}}\left(\frac{w_{j}}{w_{j-1}+w_{j}}u_{1}^{y}F_{1}^{y}+\frac{w_{j}}{w_{j+1}+w_{j}}u_{2}^{y}F_{2}^{y}\right)$$

(13) $$G_{II}=\frac{1}{2\Delta a\cdot w_{j}}\left(\frac{w_{j}}{w_{j-1}+w_{j}}u_{1}^{x}F_{1}^{x}+\frac{w_{j}}{w_{j+1}+w_{j}}u_{2}^{x}F_{2}^{x}\right)$$

(14) $$G_{III}=\frac{1}{2\Delta a\cdot w_{j}}\left(\frac{w_{j}}{w_{j-1}+w_{j}}u_{1}^{z}F_{1}^{z}+\frac{w_{j}}{w_{j+1}+w_{j}}u_{2}^{z}F_{2}^{z}\right)$$

where $F_{m}^{n}$ and $u_{m}^{n}$ (*m* = 1, 2, …, *n* = *x*, *y*, *z*) are the nodal forces and the corresponding virtual displacements along different directions on different nodes, and ∆*a* and *w_j_* are the length and width of the elements, respectively. To implement this method into Abaqus, a fracture criterion type of fatigue was edited in the Keywords module, and the direct cyclic analysis was used.

## 3. Fatigue Test and Numerical Model

### 3.1. Description of the Fatigue Test

**Figure 4:**
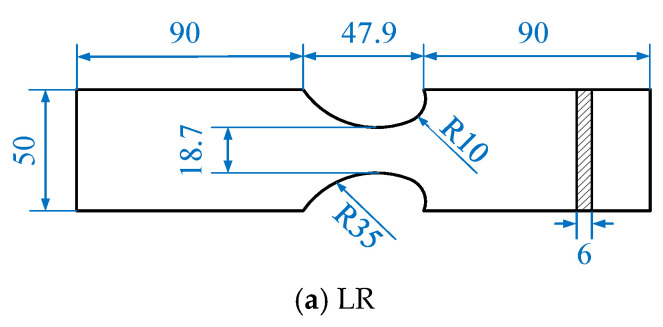

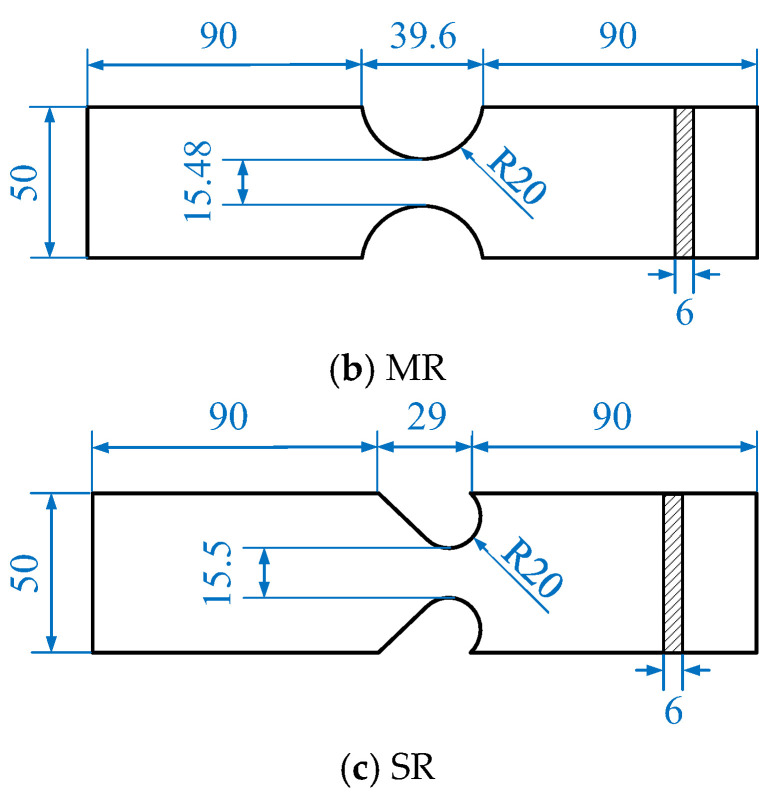
Geometries of three types of specimens (mm).

The notched specimens used in this study are shown in Figure 4. The notches in the specimens are designed based on the structural details in real bridges, and the details can be found in the authors’ recent publication [32].

**Figure 5:**
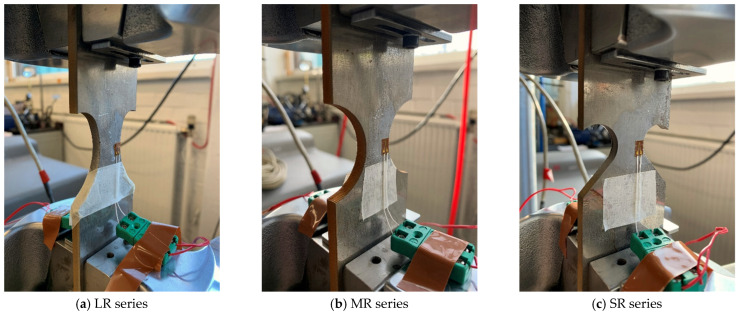
Setup of the fatigue test.

Figure 5 shows the setup of the fatigue test, and the fatigue testing machine MTS 810 (loading capacity is 100 kN) was used. For each specimen, a constant amplitude sinusoidal loading was applied with a load ratio of *R* = 0.1. The loading frequency was 20 Hz before the fatigue crack initiation, which was defined as when the peak displacement value increases to 1.25 times the initial peak displacement, was observed. After the fatigue crack initiation, the fatigue loading was terminated and then restarted with a frequency of 3 Hz. Generally, it takes another 2000 to 8000 cycles till the final fracture of the specimen. The fatigue test results of the notched specimens can be found in [32].

### 3.2. Constitutive Model of Steel Plate

Equation (15), summarized by Ramberg and Osgood [33], is used in this study to represent the constitutive relationship of the structural steel.

(15) $$\frac{\varepsilon }{2}=\frac{\sigma }{2E}+K{\left(\frac{\sigma }{2E}\right)}^{\frac{1}{n}}$$

where *K* and *n* are the material constants. When the yield strength *σ_y_* is known, Equation (15) can be reformed to Equation (16), where 1/*n* is 13.661 in this study.

(16) $$\varepsilon =\frac{\sigma }{E}+0.002{\left(\frac{\sigma }{\sigma_{y}}\right)}^{\frac{1}{n}}$$

Table 3 shows the results of the material tests of three samples.

**Figure 6:**
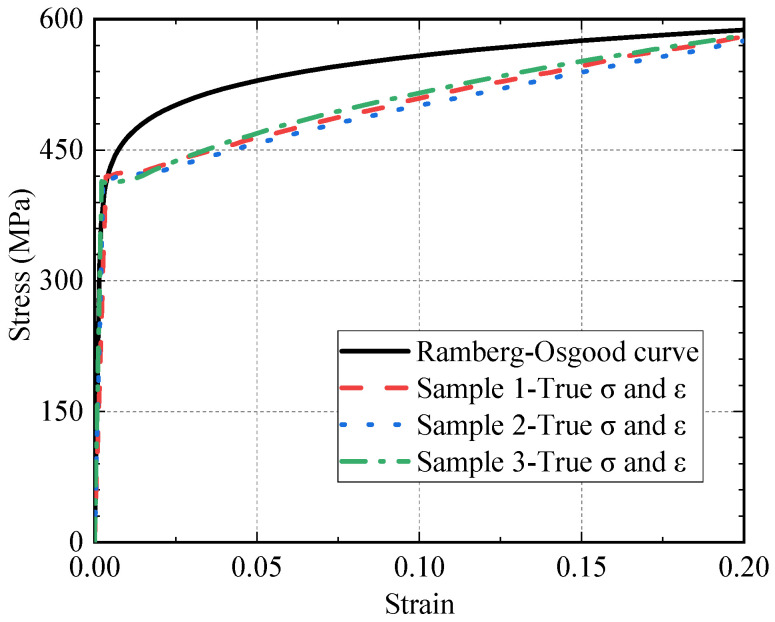
Comparison of applied constitutive model and sample tests.

Figure 6 shows the measured stress–strain curves of the steel plates that were used for the fatigue test, and the values of stress and strain have been transferred to true stress and strain, respectively. The fitted Ramberg–Osgood curve is also plotted.

As reported in [32], ratcheting behavior near the notch root was observed. To simulate such a phenomenon, the kinematic hardening of steel is used in the numerical analyses.

### 3.3. Element and Load/Boundary Conditions

**Figure 7:**
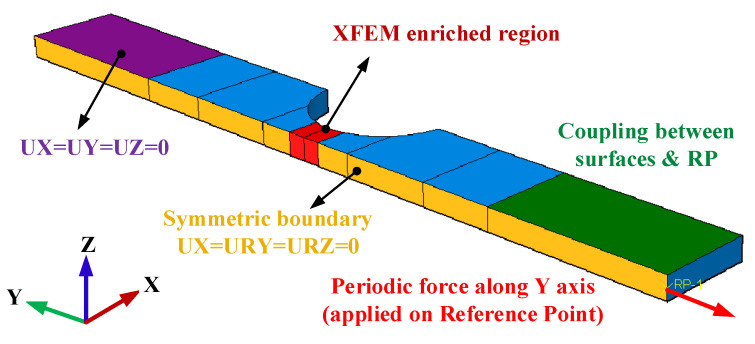
Numerical model.

To reduce the computational cost, a half-model is used, and a symmetrical boundary is imposed on the plane of symmetry, as shown in Figure 7. The model is loaded by a periodic force on a reference point (RP) which is coupled on all degrees of freedom with the clamped areas in the experimental test, and the force amplitude curve can be expressed as *a* = 0.55 + 0.45 × sin 2*πt* with a time period *T* of 1. Since the period of the curve is a unit time 1, the total step time exactly indicates the total loading cycles. To balance the computational cost and accuracy, a maximum time increment of 0.1 is set. The element type used in the model is C3D8. For the fatigue critical region where the fatigue crack is most likely to initiate, XFEM-enriched elements are assigned for capturing the crack initiation and enabling crack propagation.

## 4. Results and Discussion

### 4.1. Fatigue Life

**Figure 8:**
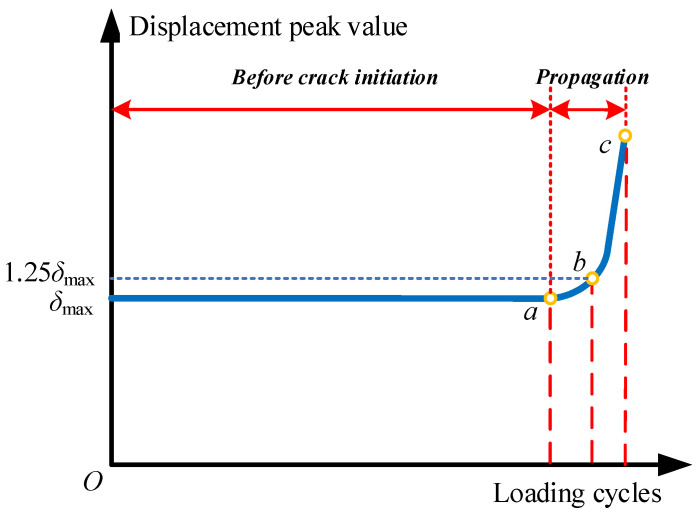
Illustration for fatigue life definition.

Concerning the definition of fatigue life, Figure 8 shows a typical evolution pattern of the peak value of displacement during a fatigue test. Before the fatigue crack initiates, the displacement peak value remains constant, while after the crack initiation, an increasing tendency can be observed, as illustrated at point *a*. In the fatigue test, the crack initiation life was determined by point *b* when the peak value of displacement reaches 1.25 times the initial value *δ*_max_ due to the lack of detecting equipment for capturing the fatigue crack initiation. As for the fatigue crack propagation life investigated in this study, the fatigue life between *a* and *c* are used to comply with the situation in numerical simulations. Since it is rather convenient to monitor the mechanical responses of numerical models, the predicted crack initiation life and propagation life in this study are defined as the loading cycles required from *O* to *a* and *a* to *c*, respectively.

Table 4 shows the comparison between the experimental and predicted fatigue crack initiation life. The error between the averaged experimental fatigue crack initiation life and predicted crack initiation life ranges from −27.5% to 41.1%. For specimens in the LR and MR series, the error decreases with the decreasing of the applied nominal-stress range, while for the SR series, the specimens with a higher nominal-stress range yield a lower error. Moreover, the produced error between the experiment and the prediction can also come from the dispersion nature of fatigue, as can be seen from the standard deviations in each case. The coefficient of variation in the fatigue tests range from 6% to 50%, indicating a relatively high extent of variability.

**Figure 9:**
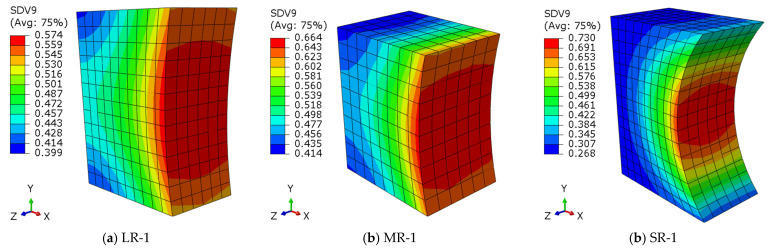
Damage contour plots at *N* = 40,000 (SDV9 = *d_f_*).

Figure 9 shows the damage contour plot when the loading cycles reach 40,000, and the SDV9, one of the nine user-defined solution-dependent state variables, is the damage factor *d_f_*. The nominal-stress ranges in LR-1, MR-1, and SR-1 are 360.96 MPa, 377.91 MPa, and 344.52 MPa, respectively. It can be seen that, as the notch root radius decreases, the distribution of the critical zone of fatigue damage is contracting towards the notch root, indicating that any initial defects located within the high-damage-factor area may result in crack initiation.

Table 5 compares the experimental and predicted fatigue crack propagation life. Different from that observed in Table 4, when the applied stress range decreases, an increase in error can be found in each series. By observing the averaged fatigue crack propagation lives within each series, it can be found that the propagation life does not change obviously with the applied stress range, and a dramatic increase occurs in the predicted propagation life. The VCCT-based crack propagation analysis is more sensitive to the change of external loads. Though there are three cases with an error of around 100%, most cases still yield acceptable results with errors ranging from −26.4% to 18.2%.

**Figure 10:**
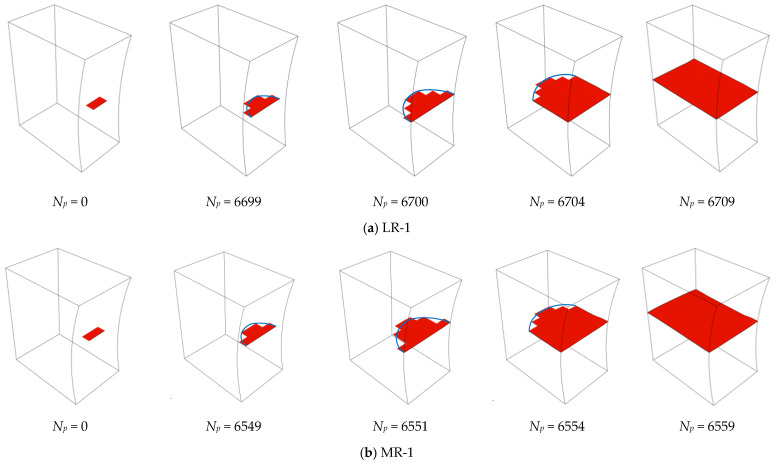

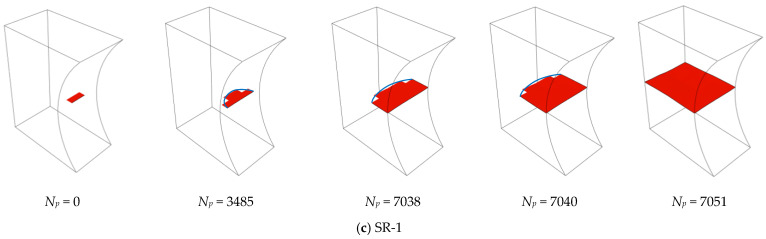
Simulated evolutions of the fatigue crack surface.

**Figure 11:**
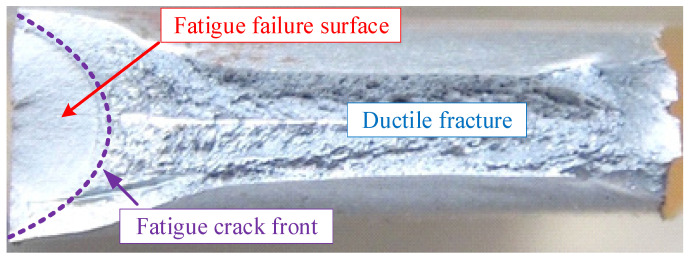
Measured fatigue crack surface in test.

Figure 10 presents the simulated evolutions of the fatigue crack surface in different series. In each series, a half-elliptical crack can be observed at the early stage of the propagation life. When the fatigue crack goes through the specimen along the thickness direction, the speed of the crack propagation dramatically accelerates due to the massive reduction of the effective area. As reported in [32], the failure modes observed in the fatigue tests are waterjet cutting edge, single plate corner, and two plate corners. The single plate corner is dominant over the other two failure modes due to the initial defects introduced by the waterjet-cutting method. After the single-plate-corner failure, the crack initiated from the cutting edge (see Figure 11) was observed more than the two plate corners. The simulated fatigue crack surface at the end of the stable propagation stage agrees well with that observed in the fatigue tests.

**Figure 12:**
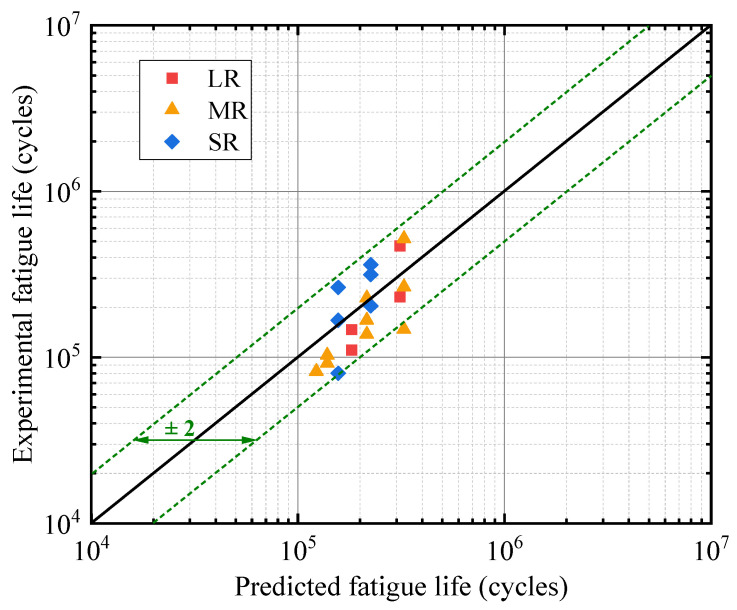
Predicted total fatigue life versus experimental life.

Figure 12 shows the comparison between the predicted total life using the proposed algorithm and experimental life (till fracture of the specimen) on a log–log scale, and the prediction has a good agreement with the experimental results with a scatter factor of around 2, which is widely accepted as a satisfactory criterion for the fatigue life prediction [34].

### 4.2. Strain Evolutions

**Figure 13:**
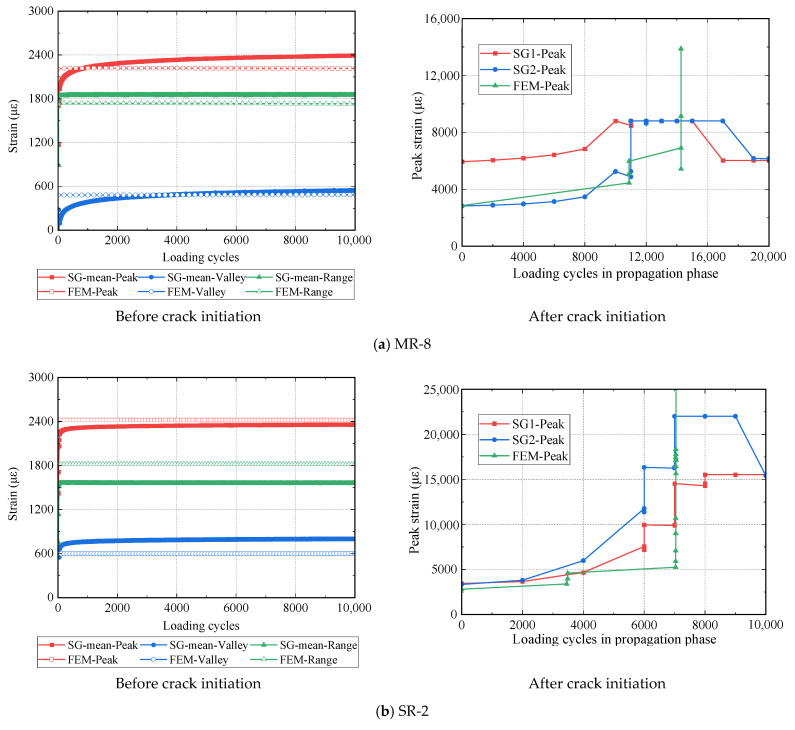

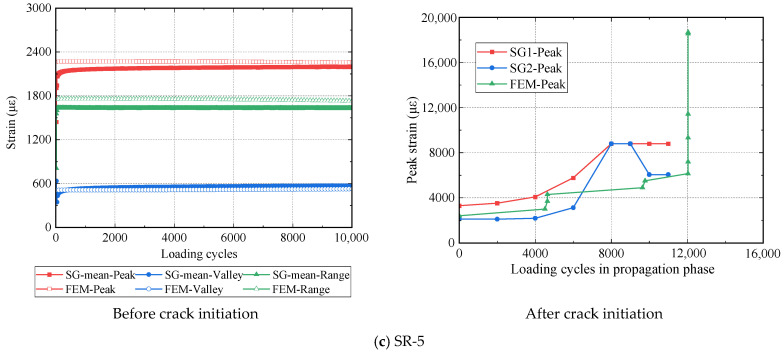
Experimental and predicted strain evolutions.

Figure 5 shows the strain evolutions in both the experimental tests and numerical simulations. Before the crack initiation, the strain in the experimental test shows a tendency to increase with the loading cycles but at a slow rate, while the strain development in numerical simulation remains stable. The measured and simulated strain values in the example specimens present minor differences with errors of less than ±20%. After the crack initiation, both strains in the experiments and simulations increase with the loading cycles. As shown in Figure 13, the simulated strain development after crack initiation has three scenarios: (1) it agrees well with one of two strain gauges that monitor the same location (MR-8); (2) it acts as a lower bound of the measurement of two strain gauges (SR-2); and (3) it is located between the measurement of two strain gauges (SR-5).

## 5. Conclusions

A new numerical model for predicting the fatigue life of different notch details was proposed in this paper, and the subroutine UDMGINI provided by Abaqus and the LEFM-based approach are employed for the prediction of fatigue crack initiation and propagation, respectively. According to the results of this study, the following conclusions are made:

(1) A new SWT-model-based algorithm for the fatigue crack initiation life prediction of notched details in the HCF regime is proposed by considering the cycle-by-cycle fatigue damage accumulation, and the damage model is implemented by employing the UDMGINI subroutine written with Fortran code. A good agreement between the predicted fatigue initiation life and experimental results is confirmed.
(2) The established numerical models based on UDMGINI and VCCT are validated and agree well with the tests, and the prediction of the total fatigue life falls within a scatter factor of 2. The failure modes predicated by the simulation are the same with the tests.
(3) Notch details with different root radii were investigated, and it was found that, as the notch root radius decreases, the high-damage-factor zone of fatigue damage becomes more concentrated, making it vulnerable to fatigue damage.

Despite the acceptable results obtained in this study, the cycle-by-cycle numerical method is time-consuming, and an optimized procedure for improving the efficiency of this method would be beneficial. In addition, the parameters used in this study were not calibrated by relevant experimental tests and were derived from the literature, and only one load ratio (0.1, i.e., tension–tension cyclic load) is validated in this study, which deserve further studies.

## Figures and Tables

**Table 1:** Material constants in SWT damage parameter of S355.

	*σ’_f_* (MPa)	*ε* *’_f_*	*b*	*c*
Cruces [29]	564.4	0.1554	−0.0576	−0.4658

**Table 2:** Material constants in Paris’ law and Abaqus.

Material	*c*_3_	*c*_4_	*G_IC_* (N/mm)	*G_IIC_* (N/mm)	*G_IIIC_* (N/mm)	*a_m_*	*a_n_*	*a_m_*
S355	1.4 × 10^−5^	1.8121	6.5	6.5	6.5	1	1	1

**Table 3:** Material properties of the structural steel.

Sample	Nominal Yield Stress (MPa)	Nominal Tensile Strength (MPa)	Elastic Modulus (GPa)	Elongation (%)
S355-1	413	476.6	208.1	45.6
S355-2	410	475.2	212.5	30.9
S355-3	414	475.7	212.2	51.6
Mean	412.3	475.8	210.9	42.7

**Table 4:** Comparison of predicted and experimental fatigue crack initiation life.

Series	Δ*σ*_nom_ (MPa)	Crack Initiation Life (Including Propagation Life Until Point *b*)						
		Sample 1	Sample 2	Sample 3	Average	Standard Deviation	XFEM	XFEM/Average
LR	360.96	110,894	147,454	--	129,174	18,280	175,077	1.355
	352.94	231,352	470,240	--	350,796	119,444	303,472	0.865
MR	377.91	82,440	--	--	82,440	--	116,326	1.411
	370.16	92,200	103,035	--	97,618	5418	130,848	1.340
	360.47	228,083	137,804	167,747	177,878	37,546	202,484	1.138
	350.78	265,791	147,792	519,846	311,143	155,239	311,628	1.002
SR	344.52	167,454	80,273	263,909	170,545	75,000	149,489	0.877
	332.90	315,173	204,465	362,291	294,043	66,153	213,190	0.725

**Table 5:** Comparison of predicted and experimental fatigue crack propagation life.

Series	Δ*σ*_nom_ (MPa)	Crack propagation					
		Sample 1	Sample 2	Sample 3	Average	XFEM	XFEM/Average
LR	360.96	8902	9333	--	9118	6709	0.736
	352.94	10,548	9534	--	10,041	8042	0.801
MR	377.91	6042	--	--	6042	6559	1.086
	370.16	7788	7560	--	7674	7785	1.014
	360.47	8241	6661	8248	7717	13,158	1.705
	350.78	9823	8799	2140	6921	14,291	2.065
SR	344.52	4945	7103	5852	5967	7051	1.182
	332.90	4112	8097	5873	6027	12,046	1.999

## Data Availability

Data are available upon request from the corresponding author.
